# The perspective of combining pulsed field ablation and left atrial appendage closure

**DOI:** 10.1093/europace/euag202

**Published:** 2026-10-21

**Authors:** Lucas Boersma, Tom De Potter, Christian-Hendrik Heeger, Devi Nair, Laurent Roten, Marco Schiavone, Claudio Tondo, Pierpaolo Iozzia, Ilaria Giabbani, Mounia Haddad, Kars Neven

**Affiliations:** Cardiology Department, St. Antonius Hospital Nieuwegein/Amsterdam University Medical Centre, Nieuwegein, the Netherlands; Cardiovascular Research Centre Aalst, AZORG Aalst, Aalst, Belgium; Department of Rhythmology, Asklepios Clinic Altona, Hamburg, Germany; Department of Rhythmology, University of Lübeck, Lubeck, Germany; Department of Cardiac Electrophysiology and Research, St. Bernard's Heart and Vascular Center, Arrhythmia Research Group, Jonesboro, AR, USA; Department of Cardiology, Inselspital, Bern University Hospital, University of Bern, Switzerland; Department of Clinical Electrophysiology and Cardiac Pacing, Centro Cardiologico Monzino, IRCCS, Milan, Italy; Department of Clinical Electrophysiology and Cardiac Pacing, Centro Cardiologico Monzino, IRCCS, Milan, Italy; Department of Surgical, Biomedical and Dental Sciences, University of Milan, Milan, Italy; Customer Education, Boston Scientific EMEA, Milan, Italy; Health Economics and Market Access, Boston Scientific EMEA, Milan, Italy; Medical Affairs, Interventional Cardiology, Boston Scientific Middle East FZ LLC, PO Box 500745, Dubai Science Park, Dubai, United Arab Emirates; Department of Electrophysiology, Alfried Krupp Hospital, Essen, Germany; Department of Medicine, Witten/Herdecke University, Witten, Germany

## Abstract

Pulsed field ablation (PFA) and left atrial appendage closure (LAAC) are increasingly discussed as complementary strategies in atrial fibrillation (AF) care, addressing rhythm control and long-term thromboembolic risk. The rationale is attractive, but the current evidence base is uneven. Randomized evidence for LAAC around AF ablation is strongest for thermal-ablation-era workflows, particularly the OPTION trial, which showed lower non-procedure-related major or clinically relevant non-major bleeding with LAAC than with oral anticoagulation (OAC) and non-inferiority for death, stroke, or systemic embolism. Dedicated PFA-specific evidence remains limited to feasibility studies and early observational experience, and no trial has demonstrated that combined ablation-LAAC procedures are clinically superior when the ablation component is performed with PFA rather than radiofrequency or cryoballoon ablation. In parallel, OAC-withdrawal trials after apparently successful ablation, including OCEAN and ALONE-AF, have reported low thromboembolic event rates in selected lower-risk populations, whereas CLOSURE-AF cautions against simple extrapolation of LAAC benefit to older, frailer patients at high stroke and bleeding risk. This review therefore positions combined PFA-LAAC as a potential option for carefully selected patients rather than an established routine strategy. We summarize procedural workflow, patient selection, trial populations, device-platform considerations, reimbursement and organizational issues, and the main uncertainties that should guide future prospective studies.

## Graphical Abstract

**Graphical Abstract:**
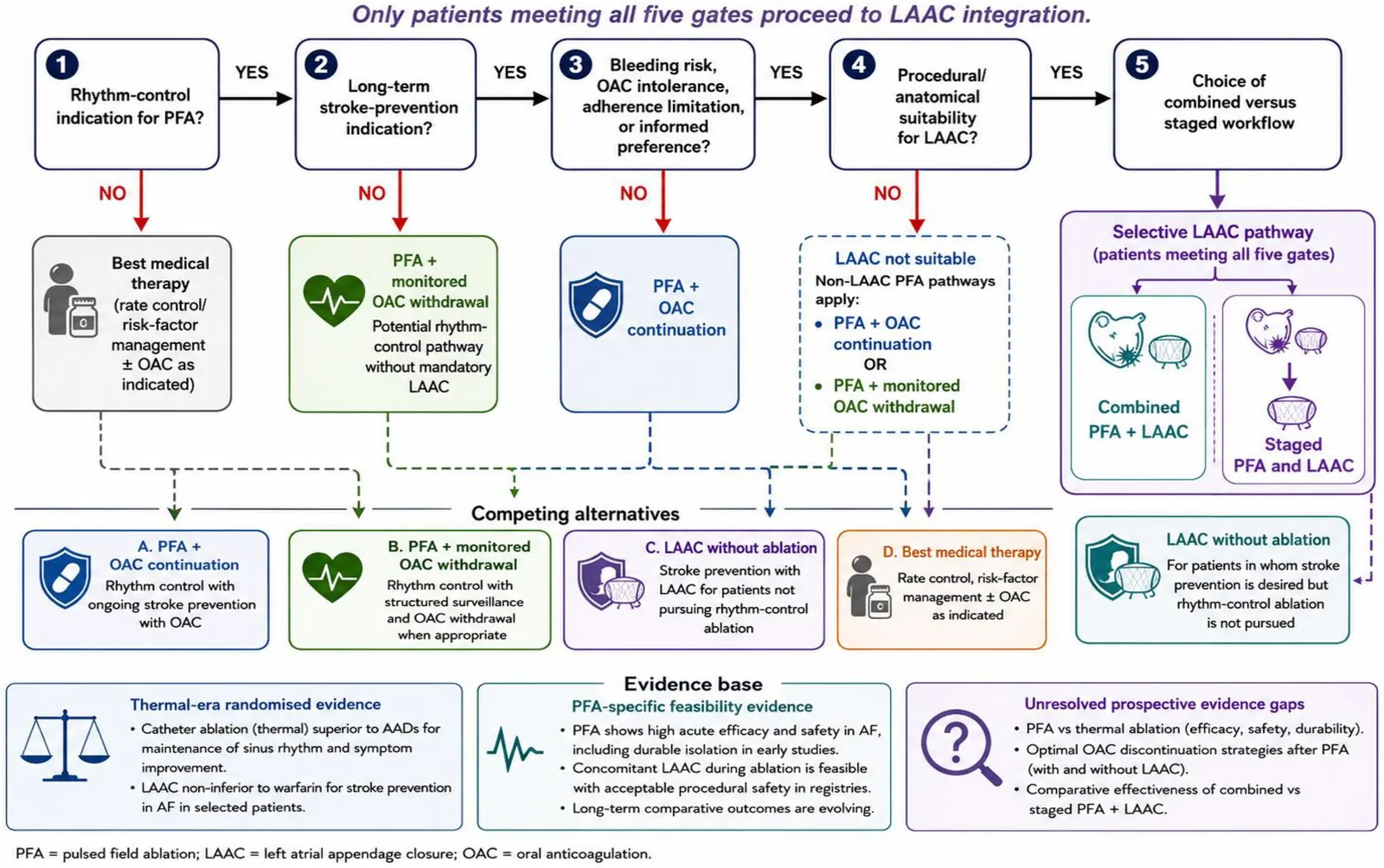
Decision framework for PFA and LAAC integration. This shows five sequential decision gates: (i) rhythm-control indication for PFA; (ii) long-term stroke-prevention indication; (iii) bleeding risk, OAC intolerance, adherence limitation, or informed preference; (iv) procedural/anatomical suitability for LAAC; and (v) choice of combined vs. staged workflow. It also highlights competing alternatives: PFA plus OAC continuation, PFA plus monitored OAC withdrawal, LAAC without ablation, and best medical therapy. Finally, it labels the evidence base as thermal-era randomized evidence, PFA-specific feasibility evidence, and unresolved prospective evidence gaps. The figure should not be interpreted as implying that PFA-LAAC is clinically superior to radiofrequency- or cryoballoon-based combined workflows.

## Why combine pulsed field ablation and left atrial appendage closure?

Catheter ablation has become a cornerstone in the management of symptomatic atrial fibrillation (AF), supported by randomized evidence showing improved rhythm control and quality of life (*Table* *1* and *2*). Advances in ablation technology have accelerated the adoption of pulsed field ablation (PFA), which uses irreversible electroporation rather than thermal injury and has demonstrated non-inferior efficacy to conventional thermal ablation in paroxysmal AF, with a favourable safety profile in large registries and contemporary consensus and review documents.^1–6^

**Table 1:** Trial populations and implications for combined PFA-LAAC

Trial	Population	Strategy	Primary endpoint(s)	Selected absolute outcomes	Relevance to PFA-LAAC
OPTION	1600 post-ablation patients; CHA_2_DS_2_-VASc ≥2 men or ≥3 women; mean CHA_2_DS_2_-VASc 3.5; low mean HAS-BLED 1.2	AF ablation plus LAAC vs. AF ablation plus OAC; ablation was RF/cryo, not PFA	Safety: non-procedure-related major or clinically relevant non-major bleeding. Efficacy: death, stroke, or systemic embolism at 36 months	Bleeding 8.5% vs. 18.1%; efficacy composite 5.3% vs. 5.8%; ischaemic stroke 1.2% vs. 1.3%	Supports LAAC as an alternative after ablation in selected patients; does not establish routine PFA-LAAC
COMBINATION	202 patients with non-valvular AF undergoing combined catheter ablation and LAAO in 14 high-volume centres; 194 completed the trial; mean age 67.3 years; 56.7% male.	Ablation-first vs. occlusion-first; contact-force RF catheter ablation and plug-like LAAO devices; not PFA-specific.	Composite of thromboembolic events including stroke or TIA, device-related thrombus, clinically relevant bleeding, and cardiovascular rehospitalization or death; rhythm outcomes.	Event-free survival 83.5% vs. 71.1% favouring occlusion-first; chronic peri-device leak 5.2% vs. 15.5%; device-related thrombus 1.0% vs. 8.2%; freedom from AF 77.3% vs. 63.5%.	Challenges a default ablation-first sequence for plug-like devices; should not be extrapolated directly to PFA-LAAC, but makes sequence a patient-, device-, and platform-specific decision.
CHAMPION-AF	3000 AF patients suitable for anticoagulation; mean age 71.7 years; mean CHA_2_DS_2_-VASc 3.5	LAAC vs. NOAC; not ablation-specific	CV death, stroke, or systemic embolism; non-procedure-related bleeding	Efficacy 5.7% vs. 4.8% (non-inferior); non-procedure-related bleeding 10.9% vs. 19.0%	Expands evidence for LAAC vs. NOAC in selected OAC-eligible patients; not PFA-specific and not an ablation trial
CLOSURE-AF	888 patients in primary analysis; older and high risk; mean age 77.9; CHA_2_DS_2_-VASc 5.2; HAS-BLED 3.0	LAAC vs. physician-directed best medical care including DOACs when feasible	Stroke, systemic embolism, major bleeding, or CV/unexplained death	Primary events 16.8 vs. 13.3 per 100 patient-years; not non-inferior	Cautions against extrapolating LAAC benefit to frail high-risk populations without careful selection
OCEAN	1284 patients at least 1 year after successful ablation; selected, no recurrent atrial tachyarrhythmia; mild-to-moderate risk	Rivaroxaban vs. aspirin; no LAAC	Stroke, systemic embolism, or new covert embolic stroke at 3 years	Primary event 0.31 vs. 0.66 per 100 patient-years; not significant; fatal/major bleeding 1.6% vs. 0.6%	Low event rates suggest OAC withdrawal may be reasonable in selected patients; not generalizable to high-risk patients
ALONE-AF	840 patients without documented recurrence at least 1 year after ablation; mean CHA_2_DS_2_-VASc 2.1	OAC discontinuation vs. continued DOAC; no LAAC	Stroke, systemic embolism, or major bleeding at 2 years	Composite 0.3% vs. 2.2%; ischaemic stroke/systemic embolism 0.3% vs. 0.8%; major bleeding 0% vs. 1.4%	Supports OAC discontinuation in carefully selected lower-risk post-ablation patients; does not remove the need for LAAC in selected higher-risk patients

AF, atrial fibrillation; CV, cardiovascular; DOAC, direct oral anticoagulant; LAA, lef atrial appendage; LAAC/LAAO, left atrial appendage closure or occlusion; NOAC, non-vitamin K antagonist oral anticoagulant; OAC, oral anticoagulation; PFA, pulsed field ablation; RF, radiofrequency; TIA, transient ischaemic attack.

**Table 2:** Disaggregated clinical interpretation of major trials

Trial	Stroke/systemic embolism	Bleeding	Mortality	Clinical interpretation
OPTION	Ischaemic stroke 1.2% vs. 1.3%; haemorrhagic stroke 0.4% vs. 0.4%; systemic embolism 0.3% vs. 0.1%	Non-procedure-related major or clinically relevant non-major bleeding 8.5% vs. 18.1%; ISTH major bleeding 3.9% vs. 5.0%	Death from any cause 3.8% vs. 4.5%	Main difference was bleeding; thromboembolic events were low in both arms.
COMBINATION	Primary composite included thromboembolic events including stroke or TIA; trial was not powered for isolated stroke/systemic embolism.	Clinically relevant bleeding was included in the composite; periprocedural complications were similar between sequence groups.	Cardiovascular rehospitalization or death was included in the composite; one non-cardiac death was reported.	Sequencing trial: occlusion-first reduced chronic peri-device leak and device-related thrombus vs. ablation-first with plug-like devices; not PFA-specific.
CHAMPION-AF	Stroke 3.6% vs. 2.5%; systemic embolism 0% vs. 0.1%	Non-procedure-related bleeding 10.9% vs. 19.0%; non-procedure-related ISTH major bleeding 5.1% vs. 6.4%	CV death 2.7% vs. 2.7%	Non-inferiority for composite efficacy with lower bleeding, but with a numerically higher stroke signal.
CLOSURE-AF	Stroke 2.6 vs. 2.7 per 100 patient-years; systemic embolism 0.3 vs. 0.1 per 100 patient-years	Major bleeding 7.4 vs. 6.2 per 100 patient-years	CV/unexplained death 9.5 vs. 7.7 per 100 patient-years	High competing risk population; no net advantage of LAAC over best medical care.
OCEAN	Stroke or systemic embolism 0.8% vs. 1.1%; new covert embolic stroke 0% vs. 0.3%	Fatal or major bleeding 1.6% vs. 0.6%; clinically relevant non-major bleeding 5.5% vs. 1.6%	All-cause death 1.6% vs. 1.1%	Low thromboembolic event rates and more bleeding with rivaroxaban in this selected population.
ALONE-AF	Ischaemic stroke/systemic embolism 0.3% vs. 0.8%; TIA 0.6% vs. 0%	Major bleeding 0% vs. 1.4%; clinically relevant non-major bleeding 1.4% vs. 1.9%	No all-cause mortality in either group	Composite advantage driven primarily by bleeding reduction.

CV, cardiovascular; ISTH, International Society on Thrombosis and Haemostasis; LAAC, left atrial appendage closure; PFA, pulsed field ablation; TIA, transient ischaemic attack.

Stroke risk after AF ablation is not determined solely by documented AF recurrence. It also reflects age, comorbidities, atrial cardiomyopathy, endothelial dysfunction, inflammation, and non- left atrial appendage (LAA) sources of embolism.^7,8^ Current guidelines therefore recommend long-term anticoagulation according to the thromboembolic risk profile rather than perceived ablation success alone.^9–11^

Oral anticoagulation (OAC) remains the standard stroke-prevention strategy in AF. Left atrial appendage closure (LAAC) is an alternative for selected patients, particularly those with an indication for stroke prevention and a contraindication to long-term OAC or unacceptable bleeding/tolerance issues. Contemporary guidance emphasizes careful referral, anatomical assessment, shared decision-making, and structured follow-up imaging after LAAC.^12^ Earlier LAAC trials and long-term follow-up established non-inferiority to warfarin in selected populations, while PRAGUE-17 provided randomized data vs. direct oral anticoagulants (DOACs) in high-risk patients.^13–15^

Combining PFA and LAAC may reduce cumulative procedural exposure by using one venous access, one transseptal access, and one anaesthetic episode. This logistical argument applies to combined ablation-LAAC in general; it does not establish a PFA-specific clinical advantage over radiofrequency or cryoballoon-based combined workflows. The concept is clinically plausible, but it should not be interpreted as proven routine care. The current task is to identify the patients for whom incremental benefit is likely to outweigh procedural risk and to distinguish evidence derived from thermal-ablation-era trials from still-limited PFA-specific data.

## Procedural workflow and technical considerations

### Patient selection

Patient selection should start with two independent questions: first, whether the patient has a clear indication for catheter ablation; second, whether LAAC is a reasonable alternative to long-term OAC. A combined procedure is most defensible when both answers are affirmative and when the risks of staging are clinically relevant. This includes patients with symptomatic AF undergoing ablation who have moderate-to-high thromboembolic risk, clinically important bleeding vulnerability, previous major or recurrent clinically relevant bleeding, OAC intolerance, difficulty maintaining long-term OAC adherence, or a strong preference to avoid lifelong OAC after informed discussion. The selection threshold should be higher in very frail patients, patients with complex LAA anatomy, active LAA thrombus, prohibitive vascular or transseptal risk, or limited life expectancy.

### Single procedure vs. staged approach

A single-procedure strategy may reduce hospital visits, repeated transseptal puncture, and repeat anaesthesia. A staged strategy remains appropriate when LAA anatomy is uncertain, when pre-procedural imaging is incomplete, when post-ablation oedema may complicate sizing, when the centre has limited combined-procedure experience, or when the stroke-prevention decision should be deferred until rhythm outcome and OAC tolerance have been reassessed. Neither strategy is intrinsically superior for all patients. Conversely, a staged strategy exposes patients to a second procedure and to an interval between ablation and LAAC during which adverse events may occur, although the available evidence for this point remains limited.^16^

### Order of procedures

When a combined approach is selected, procedural sequence should be treated as an unresolved and device-specific issue rather than as a fixed rule. In many contemporary PFA-LAAC workflows, operators perform PFA before LAAC because this allows unrestricted left atrial catheter manipulation and avoids delivering PFA close to a newly implanted LAA device. However, the COMBINATION randomized clinical trial, which compared ablation-first with occlusion-first strategies in patients undergoing combined catheter ablation and left atrial appendage closure (LAAO), reported higher event-free survival, lower chronic peri-device leak, and less device-related thrombus with an occlusion-first strategy using plug-like LAAO devices.^17^ This evidence should be reconciled with PFA-specific practice because COMBINATION was not a PFA trial, used contact-force radiofrequency ablation, and may not be directly generalizable to all LAAO devices or PFA platforms. Contemporary PFA statements note that combined PFA and LAAO is feasible but emphasize that routine use remains controversial.^18,19^ Therefore, sequence selection should consider expected ablation targets, LAA anatomy, device type, likelihood of post-ablation oedema, and whether further PFA delivery near an implanted device would be required. A PFA-first approach may be reasonable when ablation near the LAA ridge or left pulmonary veins is anticipated and no device is yet present; an occlusion-first approach may be considered when LAA sealing and device stability are the dominant concerns and further PFA close to the device can be avoided.

### Transseptal access and imaging

A single transseptal puncture is usually sufficient. The puncture should support pulmonary vein access and coaxial access to the LAA. Transoesophageal echocardiography (TEE) and intracardiac echocardiography (ICE) are both used for transseptal guidance, device sizing, deployment, leak assessment, and pericardial effusion screening. The imaging modality influences anaesthesia strategy: TEE often favours general anaesthesia or deep sedation, whereas ICE may facilitate non-general anaesthesia workflows in experienced centres.

### Pulsed field ablation-related oedema and sizing

Pulsed field ablation may produce acute atrial tissue oedema. If oedema is present or suspected, post-ablation measurements may underestimate the true LAA landing-zone dimensions after oedema resolves. For combined procedures, operators should assess LAA dimensions before ablation when feasible, integrate pre-procedural computed tomography (CT) or pre-ablation high-quality echocardiographic measurements with intraprocedural imaging, and avoid relying on a single post-ablation measurement when anatomy is borderline. Within manufacturer-specific instructions for use, operators should avoid undersizing and consider an appropriately larger or oversized device when clinically justified to reduce the risk of late peri-device leak or device instability. This should not be interpreted as evidence that PFA solves sizing or sealing limitations related to LAAC; late peri-device leak and device stability remain important follow-up endpoints.

### Interaction with existing intracardiac material

Available data and consensus statements suggest that PFA can generally be performed safely in patients with intracardiac devices, but ablation immediately adjacent to device leads or LAAO devices should be avoided where possible. Electroporation-related interaction should be regarded as a broader device-field interaction concern for any implanted LAAO device, not as a phenomenon limited to lobe-and-disc platforms. Device geometry may modulate risk, and disc protrusion may influence catheter access or local field distribution, but visible arcing has also been reported during PFA applications delivered near an implanted LAAO device.^18–20^ Pulsed field ablation should therefore not be delivered in direct contact with, or immediately adjacent to, any implanted LAAO device when avoidable. These observations support careful device-specific planning and reinforce the need to choose the procedural sequence so that PFA applications near the LAA ridge, left pulmonary veins, or an implanted LAAO device are minimized.

### Post-procedural follow-up

Post-procedural antithrombotic therapy should follow device-specific recommendations, local protocols, and patient bleeding risk. Follow-up imaging with TEE, cardiac CT, or both is needed to confirm device position, exclude device-related thrombus, and assess peri-device leak. Follow-up should also include rhythm surveillance, because LAAC does not treat AF recurrence and because stroke mechanisms after ablation are not exclusively LAA-mediated.

## Clinical evidence: separate thermal-era evidence from pulsed field ablation-specific evidence

### Historical and registry experience with combined ablation and left atrial appendage closure

Combined AF ablation and LAAC was first evaluated mainly with radiofrequency or cryoballoon ablation and early-generation LAAC devices. Single-centre and multicentre observational studies, as well as broader analyses of LAAC combined with other cardiac procedures, demonstrated procedural feasibility and generally low annual stroke rates after combined procedures.^21–24^ These studies were clinically important but non-randomized, often heterogeneous in ablation technique, device type, antithrombotic regimen, and follow-up intensity. Registry experience with contemporary WATCHMAN FLX and Amulet devices is reassuring, but observational comparisons are vulnerable to selection bias.^25,26^

### OPTION: the strongest post-ablation left atrial appendage closure evidence, but not pulsed field ablation-specific

OPTION randomized 1600 patients with AF undergoing catheter ablation and an elevated CHA_2_DS_2_-VASc score to LAAC or OAC. At 36 months, LAAC reduced non-procedure-related major or clinically relevant non-major bleeding (8.5% vs. 18.1%; hazard ratio 0.44) and was non-inferior for death, stroke, or systemic embolism (5.3% vs. 5.8%). International Society on Thrombosis and Haemostasis (ISTH) major bleeding, including procedure-related bleeding, was numerically lower with LAAC (3.9% vs. 5.0%) and met non-inferiority but not superiority. Ischaemic stroke was uncommon and similar (1.2% vs. 1.3%), as was haemorrhagic stroke (0.4% in both groups).^27^

The interpretation of OPTION should be precise. The trial supports LAAC as an alternative to OAC in selected post-ablation patients, but it does not prove superiority for individual thromboembolic endpoints, and it did not use PFA. OPTION enrollment occurred in a thermal-ablation-era workflow. Therefore, its results can inform but not validate combined PFA-LAAC as a routine strategy. Timing and rhythm sub-analyses are hypothesis-generating and support procedural feasibility, but dedicated PFA studies remain required.^28,29^

### Pulsed field ablation-specific combined evidence

Pulsed field ablation-specific evidence for concomitant LAAC is currently limited. Early feasibility studies of PFA with concomitant LAAC and single-centre experience suggest that workflows are technically feasible and that acute safety is acceptable in experienced centres.^30,31^ However, these data are not powered for stroke, systemic embolism, death, major bleeding, device-related thrombus, or long-term peri-device leak. They also do not demonstrate that combined procedures are clinically superior when performed with PFA rather than radiofrequency or cryoballoon ablation. The manuscript therefore treats PFA-LAAC as an emerging strategy rather than an established standard.

### Competing strategies: oral anticoagulation withdrawal, left atrial appendage closure without ablation, and high-risk medical therapy

OCEAN and ALONE-AF challenge the assumption that all successfully ablated patients necessarily need either lifelong OAC or LAAC. In OCEAN, 1284 patients at least 1 year after successful AF ablation were randomized to rivaroxaban or aspirin. Rivaroxaban did not significantly reduce stroke, systemic embolism, or new covert embolic stroke compared with aspirin; stroke or systemic embolism occurred in 0.8% vs. 1.1%, while fatal or major bleeding occurred in 1.6% vs. 0.6%.32 In ALONE-AF, discontinuing OAC in 840 patients without documented recurrence after ablation reduced the composite of stroke, systemic embolism, and major bleeding compared with continuing OAC (0.3% vs. 2.2% at 2 years), driven largely by bleeding; ischaemic stroke or systemic embolism remained rare (0.3% vs. 0.8%).^32^

These OAC-withdrawal studies enrolled selected patients, generally with lower thromboembolic risk than many LAAC candidates, and used protocolized rhythm surveillance. They should not be extrapolated to patients with high CHA_2_DS_2_-VA scores, limited monitoring, recurrent AF, or substantial atrial cardiomyopathy. Conversely, CLOSURE-AF cautions that LAAC benefit should not be assumed in frail, older, high-risk populations. In CLOSURE-AF, patients had a mean age of 77.9 years, a mean CHA_2_DS_2_-VASc score of 5.2, and a mean HAS-BLED score of 3.0; LAAC was not non-inferior to physician-directed medical therapy for the composite of stroke, systemic embolism, major bleeding, or cardiovascular/unexplained death.^33^

## Pulsed field ablation, anticoagulation, and the residual-risk problem

Pulsed field ablation may improve ablation safety and efficiency, but it does not by itself define long-term antithrombotic strategy and should not be assumed to improve outcomes of combined procedures beyond what has been shown for ablation-LAAC workflows in general. The central unresolved question is whether better rhythm outcomes will meaningfully reduce thromboembolic risk enough to change the threshold for OAC continuation or LAAC. Stroke after AF ablation may still occur because of silent recurrence, atrial cardiomyopathy, vascular disease, left atrial mechanical dysfunction, or non-cardioembolic mechanisms.^7,8^

Guidelines remain conservative: in patients with a long-term stroke-prevention indication, OAC decisions after ablation should be based on thromboembolic risk rather than rhythm status alone.^9–11^ Newer trials suggest that this approach may be too broad for selected lower-risk patients with durable rhythm control and rigorous monitoring, but they do not yet define a universal off-OAC strategy.^32,34^

Combined PFA-LAAC may therefore be most relevant for patients in whom ablation is clinically indicated and long-term OAC is problematic, not for all patients undergoing PFA. A patient with low thromboembolic risk and excellent rhythm control may not need LAAC. A patient with very high stroke risk, advanced frailty, and high procedural risk may be better served by optimized medical therapy. The gap between these extremes is where shared decision-making and future evidence matter most.

## Practical patient-selection framework

A pragmatic selection algorithm can be framed as sequential gates rather than a binary rule. This framework should be adapted to local guidelines, reimbursement restrictions, and patient preference (refer to *Graphical Abstract*).

**Table:** 

Decision gate	Clinical indicators favouring progression	Clinical consequence
Gate 1: Is ablation indicated?	Symptomatic AF despite medical therapy, first-line ablation in appropriate patients, tachycardia-mediated cardiomyopathy, or patient-centred rhythm-control indication.	No combined procedure if ablation itself is not justified.
Gate 2: Is stroke prevention needed?	CHA_2_DS_2_-VA score indicating long-term stroke prevention; atrial cardiomyopathy or prior stroke may strengthen concern.	Low-risk patients generally do not require LAAC.
Gate 3: Is long-term OAC problematic?	Prior major bleeding, recurrent clinically relevant bleeding, high bleeding risk not easily modifiable, intolerance, adherence problems, renal impairment affecting OAC use, or strong informed preference.	If OAC is well tolerated and bleeding risk is low, incremental LAAC benefit may be smaller.
Gate 4: Is procedural risk acceptable?	Favourable LAA anatomy, no active LAA thrombus, acceptable vascular and transseptal risk, sufficient life expectancy, centre expertise.	Avoid or stage LAAC in frail patients with high periprocedural risk or uncertain anatomy.
Gate 5: Combined or staged?	Combined if both procedures are clearly indicated, imaging is adequate, and centre workflow is mature. Staged if anatomy, oedema, antithrombotic plan, or patient preference requires reassessment.	Combined PFA-LAAC should be a selected pathway, not default practice.

## Sex-specific considerations

To date, combined ablation-LAAC studies have not demonstrated a consistent sex-specific difference in the performance of the combined strategy itself. However, available trials and registries were not primarily powered for sex-stratified assessment of procedural outcomes such as device-related thrombus, peri-device leak, stroke, bleeding, or mortality. This is relevant because trial eligibility thresholds and thromboembolic-risk schemes often differ by sex, whereas procedural endpoints are usually reported for the overall cohort. Future studies should therefore pre-specify sex-stratified analyses and report device, imaging, and antithrombotic outcomes separately for women and men.

## Device landscape and cross-platform limitations

The evidence base should not be generalized across devices without qualification. Pulsed field ablation systems differ in catheter geometry, electrode configuration, waveform, dosing logic, mapping integration, and susceptibility to safety interruptions. Left atrial appendage closure devices differ in landing zone, sealing mechanism, protrusion into the LAA ostium, anti-thrombotic regimen, and imaging requirements. These differences matter when combining procedures. In particular, apparent procedural advantages of one PFA platform or one LAAO design should not be extrapolated to other systems without platform-specific evidence.

**Table:** 

Device/domain	Examples or categories	Combined-procedure considerations	Evidence caveat
PFA platforms	Multi-spline/pentaspline catheters, circular catheters, balloon systems, focal solid-tip catheters, lattice-tip or large-footprint catheters, dual-energy PFA/RF systems	Lesion geometry, catheter manipulation, remapping workflow, sheath exchanges, and interaction with LAA devices differ by platform.	OPTION did not include PFA; early PFA-LAAC studies should not be extrapolated across all PFA systems, and no comparative evidence has shown clinical superiority of PFA-LAAC over RF- or cryoballoon-based combined workflows.
Endocardial plug-type LAAC	WATCHMAN FLX/FLX Pro and related plug-type systems	Designed to sit within the appendage ostium/landing zone; may reduce disc protrusion into the left atrium but does not remove the possibility of device-field interaction. PFA delivery in direct contact with or immediately adjacent to any implanted LAAO device should be avoided where possible.	Most randomized post-ablation LAAC evidence is WATCHMAN-based. Visible arcing has been reported near an implanted LAAO device, so apparent geometry advantages should not be over-interpreted.
Lobe-and-disc LAAC	Amulet and related lobe-and-disc systems	Disc protrusion may influence catheter access, PFA field distribution, or system safety interruption near left pulmonary veins in some anatomies. The same avoidance principle applies: do not deliver PFA close to any implanted LAAO device when avoidable.	Encouraging registry data exist, but randomized post-ablation evidence is less extensive than for WATCHMAN-based OPTION; device-field interaction concerns are not exclusive to disc-based devices.
Epicardial/surgical LAA exclusion	AtriClip and surgical/epicardial approaches	Different procedural pathway, imaging follow-up, and antithrombotic assumptions; not directly comparable with percutaneous LAAC.	Not a substitute evidence base for percutaneous concomitant PFA-LAAC.

## Reimbursement challenges and opportunities in Europe

### Policy, funding, and organizational considerations

Despite growing evidence around the safety and feasibility of the concomitant approach, the lack of supporting funding frameworks and policies can significantly delay the integration of concomitant procedures into routine clinical practice. In Europe, reimbursement decisions are decentralized and influenced by national and, in some cases, regional health technology assessments (HTAs), heterogeneous reimbursement frameworks, and payer decisions,^35^ which determine how hospitals and clinicians are remunerated and often result in fragmented implementation and heterogeneous patient access.^35^

The flexibility of systems to accommodate the concomitant approach varies widely,^36,37^ and financial sustainability may require intervention at various levels, ranging from reimbursement mechanisms to HTA processes and national policy decisions. In some countries, a critical element in adopting combined LAAC and AF ablation is defining it as a unified clinical entity within existing procedural coding systems. While AF ablation and LAAC each have established codes, some European coding systems require adjustments for their simultaneous use. When it comes to funding, in many diagnosis-related group (DRG)-based systems, reimbursement is limited to the dominant procedure per hospital admission, with the second one, often involving a high-cost device, adding substantial unreimbursed expense. Countries operating under DRGs, global budgets, fee-for-service, or mixed models may require alternative mechanisms to align incentives for concomitant procedures, ranging from the establishment of dedicated procedural codes or DRGs to add-on payments for devices or other targeted funding mechanisms.

Dedicated HTAs that assess the combined approach may also support adoption in selected countries. However, HTA outcomes are not always binding for reimbursement policy,^38^ and funding decisions may also be influenced by statements from national commissioners (e.g. NHS England), inclusion into Health Benefit Catalogues, or national clinical guidance (e.g. issued by NICE in the UK, the Haute Autorité de Santé in France, and Socialstyrelsen in Sweden). National policy, consensus documents, or clinical guidance could support the combined approach by clarifying key aspects such as funding eligibility and patient selection. Increased adoption of concomitant procedures may also pose organizational questions that should be addressed, including operator training, multidisciplinary coordination, and cath-lab and anaesthesia capacity.

### Current international reimbursement landscape

While in the United States, concomitant LAAC and AF ablation reimbursement has been introduced in 2024,^39^ reimbursement across Europe remains fragmented. In Switzerland, a DRG for concomitant AF ablation and LAAC was introduced at the beginning of 2026,^40^ but other DRG-based systems, such as Germany or Italian private accredited hospitals, reimburse one procedure per hospital admission, creating economic disincentives for concomitant rather than staged interventions. Other DRG-based systems, such as France and England, allow add-on payments for AF ablation and/or LAAC devices, which partially offsets, but does not fully eliminate, the financial disincentives for concomitant procedures. Global budget systems, such as Spain or Italian public hospitals, allow discretionary allocation of funds to cover all hospital activity, including concomitant procedures, while other European countries, such as Belgium and Croatia, have mixed reimbursement systems in which devices are funded separately from procedural costs. In many European countries, reimbursement for LAAC is restricted to patients with contraindications to long-term oral anticoagulation, thereby excluding a substantial proportion of patients enrolled in the OPTION trial.

Therefore, across Europe, the integration of concomitant LAAC and AF ablation into routine clinical practice will depend not only on the accumulation of further clinical evidence but also on reimbursement pathways and national policies evolving to reassess and reflect the value of combined interventions. Clear reimbursement frameworks, as seen in the US, can accelerate the adoption of integrated AF management strategies.

## Limitations and uncertainties

The current evidence base has important limitations. First, the strongest randomized post-ablation LAAC evidence comes from OPTION, which used radiofrequency and cryoballoon ablation rather than PFA. Second, PFA-specific combined data are early and underpowered for clinical endpoints. Third, no comparative study has shown that combined ablation-LAAC procedures are clinically superior when performed with PFA rather than radiofrequency or cryoballoon ablation. Fourth, trial populations differ substantially: OPTION enrolled post-ablation patients with relatively low bleeding risk, CHAMPION-AF enrolled OAC-eligible patients not specifically linked to ablation, CLOSURE-AF enrolled older, high-risk patients with major competing risks, and OCEAN/ALONE-AF enrolled selected patients without documented recurrence after ablation. Fifth, composite endpoints can obscure clinically important differences between stroke, bleeding, and mortality. Sixth, device-platform differences limit generalization across PFA systems and LAAC devices. Seventh, sex-specific analyses are limited and usually underpowered, despite sex-related differences in trial eligibility thresholds and thromboembolic-risk scoring. Finally, cost-effectiveness and reimbursement assumptions are country-specific and likely to change as technology, trial evidence, and payment systems evolve.

## Future perspectives

Future studies should evaluate combined PFA-LAAC with device-neutral, platform-specific reporting. At minimum, prospective studies should report ablation technology, LAAC device type, imaging modality, procedure sequence, transseptal strategy, acute complications, device-related thrombus, peri-device leak, rhythm monitoring intensity, OAC discontinuation protocol, and disaggregated clinical endpoints, including sex-stratified outcomes. Trials should compare combined PFA-LAAC not only with ablation plus continuous OAC but also with ablation plus structured OAC withdrawal or monitoring-guided OAC re-initiation. Comparative studies should also test whether PFA-LAAC offers any clinical advantage over radiofrequency- or cryoballoon-based combined workflows rather than assuming benefit from procedural efficiency or the safety profile of PFA alone.

CHAMPION-AF has now reported that LAAC was non-inferior to non-vitamin K antagonist oral anticoagulant (NOAC) therapy for cardiovascular death, stroke, or systemic embolism and reduced non-procedure-related bleeding in OAC-eligible patients.^41^ This supports a broader discussion of LAAC as a potential alternative to NOACs in selected patients, but it does not answer the ablation-specific or PFA-specific question. CLOSURE-AF simultaneously reminds the field that high-risk frail patients may have competing risks that can offset expected LAAC benefit.^33^ Together, these trials argue for more precise patient selection rather than universal expansion.

## Conclusions

Combined PFA and LAAC is a technically plausible and increasingly relevant strategy for selected patients with AF who require rhythm control and have a clinically meaningful reason to avoid long-term OAC. Current randomized evidence supports LAAC around AF ablation mainly from thermal-ablation-era data, while PFA-specific evidence remains limited and does not yet establish superiority over radiofrequency- or cryoballoon-based combined procedures. The strategy should therefore be presented as an emerging, patient-selected pathway requiring prospective validation, not as routine standard care.

## Data Availability

The data underlying this article will be shared on reasonable request to the corresponding author.

## Final approval of manuscript

All authors.
